# 1,10-Phenanthrolin-1-ium 2-carb­oxy-4,5-dichloro­benzoate

**DOI:** 10.1107/S1600536809034448

**Published:** 2009-09-05

**Authors:** Graham Smith, Urs D. Wermuth, Jonathan M. White

**Affiliations:** a School of Physical and Chemical Sciences, Queensland University of Technology, GPO Box 2434, Brisbane, Queensland 4001, Australia; b BIO-21 Molecular Science and Biotechnology, University of Melbourne, Parkville, Victoria 3052, Australia

## Abstract

In the structure of the 1:1 proton-transfer compound of 1,10-phenanthroline with 4,5-dichloro­phthalic acid, C_12_H_9_N_2_^+^·C_8_H_3_Cl_2_O_4_^−^, determined at 130 K, the 1,10-phenanthrolinium cation and the hydrogen 4,5-dichloro­phthalate anion associate through a single N—H⋯O_carbox­yl_ hydrogen bond giving discrete units which have no extension except through a number of weak cation C—H⋯O_anion_ associations and weak cation–anion aromatic ring π–π inter­actions [minimum centroid–centroid separation = 3.6815 (12) Å]. The anions are essentially planar "[maximum deviation 0.214 (1) Å (a carboxyl O)] with the *syn*-related H atom of the carboxyl group, forming a short intra­molecular O—H⋯O_carbox­yl_ hydrogen bond.

## Related literature

For the structures of other hydrogen 4,5-dichloro­phthalate salts, see: Mallinson *et al.* (2003 ▶); Bozkurt *et al.* (2006 ▶); Smith *et al.* (2007 ▶, 2008*a* ▶,*b* ▶, 2009*a* ▶,*b* ▶). For hydrogen-bond motifs, see: Etter *et al.* (1990 ▶).
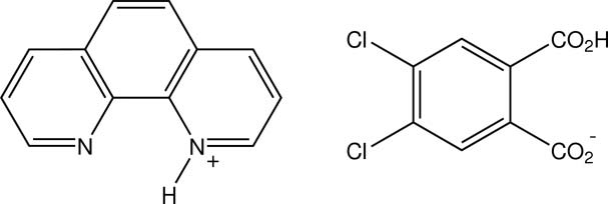


## Experimental

### 

#### Crystal data

C_12_H_9_N_2_^+^·C_8_H_3_Cl_2_O_4_^−^
*M* *_r_* = 415.22
Monoclinic, 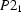

*a* = 6.4598 (11) Å
*b* = 7.3696 (12) Å
*c* = 18.302 (3) Å
β = 94.978 (3)°
*V* = 868.0 (2) Å^3^
*Z* = 2
Mo *K*α radiation
μ = 0.41 mm^−1^
*T* = 130 K
0.55 × 0.45 × 0.05 mm

#### Data collection

Bruker SMART CCD area-detector diffractometer
Absorption correction: multi-scan (**SADABS**; Sheldrick, 1996 ▶) *T*_min_ = 0.81, *T*_max_ = 0.98
5464 measured reflections
3734 independent reflections
3629 reflections with *I* > 2σ(*I*)
*R*_int_ = 0.017

#### Refinement

*R*[*F* ^2^ > 2σ(*F* ^2^)] = 0.032
*wR*(*F* ^2^) = 0.085
*S* = 1.04
3734 reflections
261 parameters
1 restraint
H atoms treated by a mixture of independent and constrained refinement
Δρ_max_ = 0.30 e Å^−3^
Δρ_min_ = −0.19 e Å^−3^
Absolute structure: Flack (1983 ▶), 1564 Friedel pairs
Flack parameter: 0.00 (4)

### 

Data collection: *SMART* (Bruker, 2000 ▶); cell refinement: *SAINT* (Bruker, 1999 ▶); data reduction: *SAINT*; program(s) used to solve structure: *SHELXS97* (Sheldrick, 2008 ▶); program(s) used to refine structure: *SHELXL97* (Sheldrick, 2008 ▶); molecular graphics: *PLATON* (Spek, 2009 ▶); software used to prepare material for publication: *PLATON*.

## Supplementary Material

Crystal structure: contains datablocks global, I. DOI: 10.1107/S1600536809034448/fl2261sup1.cif

Structure factors: contains datablocks I. DOI: 10.1107/S1600536809034448/fl2261Isup2.hkl

Additional supplementary materials:  crystallographic information; 3D view; checkCIF report

## Figures and Tables

**Table 1:** Hydrogen-bond geometry (Å, °)

*D*—H⋯*A*	*D*—H	H⋯*A*	*D*⋯*A*	*D*—H⋯*A*
N1*A*—H1*A*⋯O22	0.90 (2)	1.83 (2)	2.6926 (19)	158 (2)
N1*A*—H1*A*⋯N10*A*	0.90 (2)	2.38 (2)	2.749 (2)	104.3 (15)
O12—H12⋯O21	0.98 (3)	1.43 (3)	2.4054 (19)	179 (4)
C2*A*—H2*A*⋯O21	0.93	2.52	3.279 (2)	140
C3—H3⋯O22	0.93	2.26	2.647 (2)	104
C3*A*—H3*A*⋯O11^i^	0.93	2.44	3.355 (2)	168
C4*A*—H4*A*⋯O21^ii^	0.93	2.49	3.252 (2)	139
C6—H6⋯O11	0.93	2.29	2.668 (2)	103
C6*A*—H6*A*⋯O11^iii^	0.93	2.59	3.270 (2)	130

Symmetry codes: (i) 
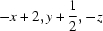
; (ii) 
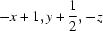
; (iii) 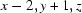.

**Fig. 1.**
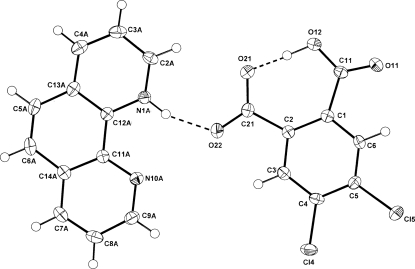
Molecular configuration and atom numbering scheme for the 1,10-phenanthrolin-1-ium cation and the hydrogen 4,5-dichlorophthalate anion in (I). Non-H atoms are shown as 50% probability displacement ellipsoids. The inter-species hydrogen bond is shown as a dashed line.

**Fig. 2.**
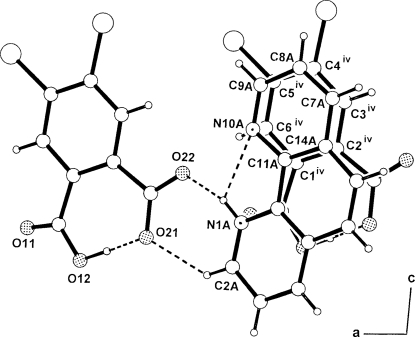
Cation–anion aromatic ring overlap in (I) viewed down the approximate b direction in the unit cell. For symmetry code (iv): x + 1, y - 1, z.

**Fig. 3.**
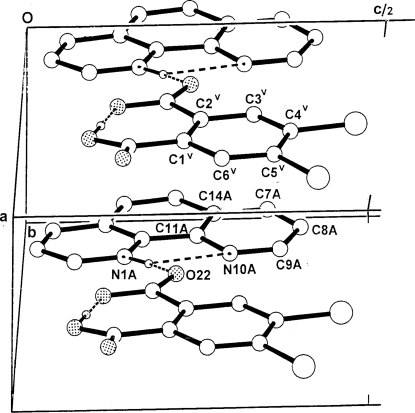
Aromatic ring π–π interactions in a perspective view of part of the unit cell. Non-interactive H atoms are omitted. For symmetry code (v): x - 1, y, z.

## supplementary crystallographic information

### Comment

The 1:1 proton-transfer compounds of 4,5-dichlorophthalic acid (DCPA) with the aromatic nitrogen Lewis bases commonly have low-dimensional hydrogen-bonded structures (Smith *et al.*, 2007, 2008a, 2008b, 2009a, 2009b; Bozkurt *et al.*, 2006; Mallinson *et al.*, 2003). In the two-dimensional examples the DCPA anions assume non-planar conformations and form into sheet substructures which in the case of the compounds with the *meta*- and *para*-aminobenzoic acids (Smith *et al.*, 2008b) are extended into three-dimensional frameworks through peripheral cyclic head-to-head carboxylic acid hydrogen-bonding associations. However, with the majority of the structures, e.g. the brucinium salt (Smith *et al.*, 2007), the DCPA anions are essentially planar with short intramolecular carboxylic acid O–H···O_carboxyl_ hydrogen bonds. These features were therefore expected and found in the 1:1 proton-transfer compound of DCPA with 1,10-phenanthroline,  (I), reported here.

In (I), a single N^+^–H···O_carboxyl_ hydrogen bond links the phenanthroline cation and the DCPA anion (Fig. 1). A weak aromatic ring C–H···O_carboxyl_ interaction (Table 1) completes an asymmetric R^2^_2_(7) cyclic association (Etter *et al.*, 1990). Three additional anion C–H···O interactions represent the only structure extensions present. Some overlap is present between the anion aromatic ring (C1–C6) and one six-membered ring of the cation (N10A, C9A, C8A, C7A, C6A, C14A) [minimum ring centroid separation, 3.6815 (12) Å] (Fig. 2), giving weak π–π stacking interactions (Fig. 3). The DCPA anion is essentially planar [torsion angles C2–C1–C11–O11, -168.30 (16)°: C1–C2–C21–O22, -179.53 (16)°], and exhibits a short intramolecular O–H···O_carboxyl_ hydrogen bond [2.4054 (19) Å]. Associated with this bond is a significant distortion of the *exo*-C1 and C2 bond angles [C1–C2–C21, 128.55 (15) ° and C2–C1–C11, 129.18 (16) °]. This and a lengthening of the C1–C11 and C2–C21 bonds [1.538 (3) and 1.536 (3) Å] is common to the planar DCPA anions in the series of 1:1 proton-transfer compounds [angle range: 127.88 (16)° in the nicotinamide salt (Smith *et al.*, 2009a) to 129.27 (14)° in the 8-aminoquinoline salt (Smith *et al.*, 2008a); bond length range: 1.523 (3)–1.535 (3) Å, both in the brucinium salt (Smith *et al.*, 2007).

### Experimental

The title compound (I) was synthesized by heating 1 mmol quantities of 1,10-phenanthroline and 4,5-dichlorophthalic acid in 50 ml of 95% ethanol for 10 min under reflux. After concentration to *ca*. 30 ml, partial room-temperature evaporation of the hot-filtered solution gave colourless plates (m.p. 464–465 K) suitable for data collection.

### Refinement

Hydrogen atoms potentially involved in hydrogen-bonding interactions were located by difference methods and their positional and isotropic displacement parameters were refined. Other H atoms were included in the refinement at calculated positions [C–H, 0.93 Å] and treated as riding models with *U*_iso_(H) = 1.2*U*_eq_ (C).

### Crystal data

**Table:**

C_12_H_9_N_2_^+^·C_8_H_3_Cl_2_O_4_^−^	*F*(000) = 424
*M**_r_* = 415.22	*D*_x_ = 1.589 Mg m^−^^3^
Monoclinic, *P*2_1_	Melting point = 464–465 K
Hall symbol: P 2yb	Mo *K*α radiation, λ = 0.71073 Å
*a* = 6.4598 (11) Å	Cell parameters from 3630 reflections
*b* = 7.3696 (12) Å	θ = 2.2–27.5°
*c* = 18.302 (3) Å	µ = 0.41 mm^−^^1^
β = 94.978 (3)°	*T* = 130 K
*V* = 868.0 (2) Å^3^	Plate, colourless
*Z* = 2	0.55 × 0.45 × 0.05 mm

### Data collection

**Table:**

Bruker SMART CCD area-detector diffractometer	3734 independent reflections
Radiation source: sealed tube	3629 reflections with *I* > 2σ(*I*)
graphite	*R*_int_ = 0.017
φ and ω scans	θ_max_ = 27.6°, θ_min_ = 1.1°
Absorption correction: multi-scan (*SADABS*; Sheldrick, 1996)	*h* = −5→8
*T*_min_ = 0.81, *T*_max_ = 0.98	*k* = −9→9
5464 measured reflections	*l* = −23→21

### Refinement

**Table:**

Refinement on *F*^2^	Secondary atom site location: difference Fourier map
Least-squares matrix: full	Hydrogen site location: inferred from neighbouring sites
*R*[*F*^2^ > 2σ(*F*^2^)] = 0.032	H atoms treated by a mixture of independent and constrained refinement
*wR*(*F*^2^) = 0.085	*w* = 1/[σ^2^(*F*_o_^2^) + (0.053*P*)^2^ + 0.0522*P*] where *P* = (*F*_o_^2^ + 2*F*_c_^2^)/3
*S* = 1.04	(Δ/σ)_max_ = 0.001
3734 reflections	Δρ_max_ = 0.30 e Å^−^^3^
261 parameters	Δρ_min_ = −0.19 e Å^−^^3^
1 restraint	Absolute structure: Flack (1983), 1564 Friedel pairs
Primary atom site location: structure-invariant direct methods	Flack parameter: 0.00 (4)

### Special details

**Table:**

Geometry. Bond distances, angles *etc*. have been calculated using the rounded fractional coordinates. All su's are estimated from the variances of the (full) variance-covariance matrix. The cell e.s.d.'s are taken into account in the estimation of distances, angles and torsion angles
Refinement. Refinement of *F*^2^ against ALL reflections. The weighted *R*-factor *wR* and goodness of fit *S* are based on *F*^2^, conventional *R*-factors *R* are based on *F*, with *F* set to zero for negative *F*^2^. The threshold expression of *F*^2^ > σ(*F*^2^) is used only for calculating *R*-factors(gt) *etc*. and is not relevant to the choice of reflections for refinement. *R*-factors based on *F*^2^ are statistically about twice as large as those based on *F*, and *R*- factors based on ALL data will be even larger.

### Fractional atomic coordinates and isotropic or equivalent isotropic displacement parameters (Å^2^)

**Table:**

	*x*	*y*	*z*	*U*_iso_*/*U*_eq_	
N1A	0.4667 (2)	0.7304 (2)	0.14863 (8)	0.0192 (4)	
N10A	0.4177 (2)	0.7715 (2)	0.29542 (8)	0.0212 (4)	
C2A	0.5046 (3)	0.7149 (3)	0.07881 (10)	0.0232 (5)	
C3A	0.3645 (3)	0.7828 (3)	0.02322 (10)	0.0277 (5)	
C4A	0.1845 (3)	0.8641 (3)	0.04213 (10)	0.0268 (5)	
C5A	−0.0459 (3)	0.9562 (3)	0.13825 (11)	0.0233 (5)	
C6A	−0.0804 (3)	0.9634 (2)	0.20961 (11)	0.0246 (5)	
C7A	0.0442 (3)	0.9113 (2)	0.34093 (10)	0.0259 (6)	
C8A	0.2013 (3)	0.8540 (3)	0.39058 (10)	0.0286 (5)	
C9A	0.3857 (3)	0.7854 (3)	0.36523 (10)	0.0253 (5)	
C11A	0.2617 (3)	0.8274 (2)	0.24641 (9)	0.0184 (4)	
C12A	0.2916 (3)	0.8115 (2)	0.16952 (10)	0.0178 (4)	
C13A	0.1421 (3)	0.8783 (2)	0.11565 (10)	0.0207 (4)	
C14A	0.0717 (3)	0.9002 (2)	0.26564 (10)	0.0207 (5)	
Cl4	1.15820 (7)	0.37080 (8)	0.46649 (2)	0.0332 (1)	
Cl5	1.59276 (7)	0.22144 (8)	0.42219 (2)	0.0316 (1)	
O11	1.5052 (2)	0.18688 (18)	0.14604 (7)	0.0251 (4)	
O12	1.2404 (2)	0.34157 (19)	0.09355 (7)	0.0265 (4)	
O21	0.9277 (2)	0.4836 (2)	0.12771 (7)	0.0271 (4)	
O22	0.76248 (19)	0.53666 (18)	0.22655 (7)	0.0254 (4)	
C1	1.2820 (3)	0.3211 (2)	0.22748 (9)	0.0179 (5)	
C2	1.0928 (3)	0.3971 (2)	0.24707 (9)	0.0178 (4)	
C3	1.0603 (3)	0.4092 (2)	0.32117 (10)	0.0206 (5)	
C4	1.2096 (3)	0.3536 (3)	0.37559 (9)	0.0215 (5)	
C5	1.3983 (3)	0.2862 (2)	0.35636 (10)	0.0207 (5)	
C6	1.4314 (3)	0.2693 (2)	0.28333 (10)	0.0196 (4)	
C11	1.3502 (3)	0.2788 (2)	0.15083 (9)	0.0195 (5)	
C21	0.9127 (3)	0.4764 (2)	0.19673 (9)	0.0193 (4)	
H1A	0.556 (3)	0.683 (3)	0.1842 (12)	0.021 (5)*	
H2A	0.62580	0.65840	0.06680	0.0280*	
H3A	0.39190	0.77330	−0.02570	0.0330*	
H4A	0.09010	0.91030	0.00560	0.0320*	
H5A	−0.14490	1.00210	0.10310	0.0280*	
H6A	−0.20540	1.01040	0.22290	0.0300*	
H7A	−0.07860	0.95670	0.35670	0.0310*	
H8A	0.18650	0.86030	0.44060	0.0340*	
H9A	0.49080	0.74780	0.39980	0.0300*	
H3	0.93520	0.45580	0.33450	0.0250*	
H6	1.55700	0.22200	0.27080	0.0230*	
H12	1.113 (5)	0.400 (4)	0.1072 (17)	0.035 (9)*	

### Atomic displacement parameters (Å^2^)

**Table:**

	*U*^11^	*U*^22^	*U*^33^	*U*^12^	*U*^13^	*U*^23^
N1A	0.0167 (6)	0.0212 (7)	0.0195 (7)	−0.0015 (6)	0.0013 (5)	0.0001 (6)
N10A	0.0217 (7)	0.0224 (7)	0.0193 (7)	0.0012 (6)	0.0005 (6)	−0.0004 (6)
C2A	0.0222 (8)	0.0261 (8)	0.0222 (8)	−0.0047 (8)	0.0078 (7)	−0.0028 (7)
C3A	0.0292 (10)	0.0360 (10)	0.0182 (8)	−0.0077 (8)	0.0036 (7)	0.0005 (7)
C4A	0.0268 (9)	0.0298 (9)	0.0230 (8)	−0.0069 (8)	−0.0029 (7)	0.0060 (8)
C5A	0.0176 (8)	0.0211 (8)	0.0302 (9)	−0.0004 (7)	−0.0040 (7)	0.0041 (7)
C6A	0.0164 (8)	0.0202 (9)	0.0375 (10)	0.0014 (7)	0.0033 (7)	−0.0017 (8)
C7A	0.0242 (9)	0.0253 (10)	0.0295 (10)	0.0011 (8)	0.0104 (7)	−0.0027 (7)
C8A	0.0358 (10)	0.0308 (10)	0.0203 (8)	0.0001 (9)	0.0096 (7)	−0.0023 (8)
C9A	0.0295 (9)	0.0263 (9)	0.0197 (8)	0.0025 (8)	−0.0007 (7)	0.0020 (7)
C11A	0.0182 (8)	0.0164 (7)	0.0207 (8)	−0.0009 (6)	0.0026 (6)	0.0009 (6)
C12A	0.0163 (7)	0.0166 (7)	0.0204 (8)	−0.0040 (6)	0.0005 (6)	0.0001 (6)
C13A	0.0186 (7)	0.0188 (7)	0.0241 (8)	−0.0039 (7)	−0.0020 (6)	0.0027 (7)
C14A	0.0195 (8)	0.0181 (8)	0.0248 (8)	−0.0031 (7)	0.0039 (6)	−0.0021 (7)
Cl4	0.0291 (2)	0.0541 (3)	0.0169 (2)	0.0014 (2)	0.0045 (2)	−0.0034 (2)
Cl5	0.0251 (2)	0.0463 (3)	0.0221 (2)	0.0067 (2)	−0.0046 (2)	−0.0001 (2)
O11	0.0227 (6)	0.0297 (7)	0.0236 (6)	0.0044 (6)	0.0058 (5)	−0.0029 (5)
O12	0.0260 (6)	0.0354 (7)	0.0187 (6)	0.0056 (6)	0.0052 (5)	0.0018 (5)
O21	0.0264 (7)	0.0343 (7)	0.0204 (6)	0.0068 (6)	0.0009 (5)	0.0030 (5)
O22	0.0200 (6)	0.0284 (7)	0.0277 (7)	0.0044 (5)	0.0023 (5)	0.0012 (5)
C1	0.0189 (8)	0.0157 (8)	0.0192 (8)	−0.0027 (6)	0.0029 (6)	0.0006 (6)
C2	0.0188 (8)	0.0149 (7)	0.0197 (8)	−0.0012 (6)	0.0024 (6)	0.0007 (6)
C3	0.0189 (8)	0.0215 (9)	0.0215 (8)	−0.0012 (7)	0.0032 (7)	−0.0034 (6)
C4	0.0229 (8)	0.0263 (9)	0.0161 (7)	−0.0035 (7)	0.0057 (6)	−0.0019 (7)
C5	0.0188 (8)	0.0231 (8)	0.0196 (8)	−0.0005 (7)	−0.0019 (6)	0.0019 (7)
C6	0.0155 (7)	0.0207 (8)	0.0229 (8)	0.0010 (6)	0.0040 (6)	−0.0014 (7)
C11	0.0202 (8)	0.0197 (8)	0.0192 (8)	−0.0041 (7)	0.0055 (6)	−0.0012 (6)
C21	0.0168 (7)	0.0173 (8)	0.0233 (8)	−0.0023 (6)	−0.0005 (6)	−0.0004 (6)

### Geometric parameters (Å, °)

**Table:**

Cl4—C4	1.7291 (17)	C11A—C12A	1.442 (2)
Cl5—C5	1.7305 (19)	C11A—C14A	1.412 (3)
O11—C11	1.218 (2)	C12A—C13A	1.408 (3)
O12—C11	1.299 (2)	C2A—H2A	0.9300
O21—C21	1.276 (2)	C3A—H3A	0.9300
O22—C21	1.236 (2)	C4A—H4A	0.9300
O12—H12	0.98 (3)	C5A—H5A	0.9300
N1A—C12A	1.363 (2)	C6A—H6A	0.9300
N1A—C2A	1.327 (2)	C7A—H7A	0.9300
N10A—C11A	1.354 (2)	C8A—H8A	0.9300
N10A—C9A	1.316 (2)	C9A—H9A	0.9300
N1A—H1A	0.90 (2)	C1—C2	1.419 (3)
C2A—C3A	1.395 (3)	C1—C11	1.538 (2)
C3A—C4A	1.379 (3)	C1—C6	1.397 (3)
C4A—C13A	1.400 (3)	C2—C21	1.536 (3)
C5A—C6A	1.345 (3)	C2—C3	1.393 (2)
C5A—C13A	1.436 (3)	C3—C4	1.387 (3)
C6A—C14A	1.435 (3)	C4—C5	1.390 (3)
C7A—C14A	1.407 (3)	C5—C6	1.377 (3)
C7A—C8A	1.369 (3)	C3—H3	0.9300
C8A—C9A	1.409 (3)	C6—H6	0.9300
			
C11—O12—H12	111 (2)	C14A—C6A—H6A	119.00
C2A—N1A—C12A	122.34 (16)	C5A—C6A—H6A	119.00
C9A—N10A—C11A	116.68 (15)	C8A—C7A—H7A	121.00
C12A—N1A—H1A	117.5 (13)	C14A—C7A—H7A	121.00
C2A—N1A—H1A	120.1 (13)	C9A—C8A—H8A	120.00
N1A—C2A—C3A	120.63 (18)	C7A—C8A—H8A	120.00
C2A—C3A—C4A	118.74 (17)	C8A—C9A—H9A	118.00
C3A—C4A—C13A	120.89 (17)	N10A—C9A—H9A	118.00
C6A—C5A—C13A	120.73 (18)	C2—C1—C11	129.18 (16)
C5A—C6A—C14A	121.35 (18)	C2—C1—C6	118.60 (15)
C8A—C7A—C14A	118.87 (17)	C6—C1—C11	112.19 (16)
C7A—C8A—C9A	119.43 (17)	C1—C2—C21	128.55 (15)
N10A—C9A—C8A	123.79 (17)	C1—C2—C3	118.51 (16)
N10A—C11A—C14A	124.31 (15)	C3—C2—C21	112.92 (16)
C12A—C11A—C14A	117.85 (16)	C2—C3—C4	121.74 (17)
N10A—C11A—C12A	117.83 (16)	Cl4—C4—C5	121.05 (14)
C11A—C12A—C13A	120.89 (17)	C3—C4—C5	119.63 (16)
N1A—C12A—C11A	119.61 (16)	Cl4—C4—C3	119.32 (15)
N1A—C12A—C13A	119.50 (16)	C4—C5—C6	119.44 (17)
C5A—C13A—C12A	118.94 (17)	Cl5—C5—C4	121.47 (14)
C4A—C13A—C5A	123.19 (17)	Cl5—C5—C6	119.09 (15)
C4A—C13A—C12A	117.87 (17)	C1—C6—C5	122.00 (17)
C6A—C14A—C7A	122.97 (17)	O11—C11—C1	118.72 (15)
C7A—C14A—C11A	116.91 (16)	O12—C11—C1	118.97 (15)
C6A—C14A—C11A	120.09 (16)	O11—C11—O12	122.32 (16)
C3A—C2A—H2A	120.00	O22—C21—C2	117.04 (15)
N1A—C2A—H2A	120.00	O21—C21—O22	123.49 (17)
C2A—C3A—H3A	121.00	O21—C21—C2	119.41 (16)
C4A—C3A—H3A	121.00	C2—C3—H3	119.00
C3A—C4A—H4A	120.00	C4—C3—H3	119.00
C13A—C4A—H4A	120.00	C1—C6—H6	119.00
C6A—C5A—H5A	120.00	C5—C6—H6	119.00
C13A—C5A—H5A	120.00		
			
C12A—N1A—C2A—C3A	0.2 (3)	N1A—C12A—C13A—C5A	177.27 (16)
C2A—N1A—C12A—C11A	−178.71 (17)	C11A—C12A—C13A—C4A	177.79 (16)
C2A—N1A—C12A—C13A	1.4 (3)	C11A—C12A—C13A—C5A	−2.6 (2)
C11A—N10A—C9A—C8A	0.0 (3)	C6—C1—C2—C3	−2.9 (2)
C9A—N10A—C11A—C12A	−179.22 (16)	C6—C1—C2—C21	175.21 (15)
C9A—N10A—C11A—C14A	0.8 (2)	C11—C1—C2—C3	174.90 (15)
N1A—C2A—C3A—C4A	−0.9 (3)	C11—C1—C2—C21	−7.0 (3)
C2A—C3A—C4A—C13A	−0.1 (3)	C2—C1—C6—C5	1.5 (2)
C3A—C4A—C13A—C5A	−177.89 (19)	C11—C1—C6—C5	−176.68 (14)
C3A—C4A—C13A—C12A	1.7 (3)	C2—C1—C11—O11	−168.30 (16)
C13A—C5A—C6A—C14A	2.4 (3)	C2—C1—C11—O12	11.4 (3)
C6A—C5A—C13A—C4A	178.76 (18)	C6—C1—C11—O11	9.6 (2)
C6A—C5A—C13A—C12A	−0.8 (3)	C6—C1—C11—O12	−170.67 (15)
C5A—C6A—C14A—C7A	177.74 (17)	C1—C2—C3—C4	1.7 (2)
C5A—C6A—C14A—C11A	−0.5 (2)	C21—C2—C3—C4	−176.64 (16)
C14A—C7A—C8A—C9A	−0.1 (3)	C1—C2—C21—O21	−2.2 (3)
C8A—C7A—C14A—C6A	−177.47 (17)	C1—C2—C21—O22	−179.53 (16)
C8A—C7A—C14A—C11A	0.8 (2)	C3—C2—C21—O21	175.96 (15)
C7A—C8A—C9A—N10A	−0.3 (3)	C3—C2—C21—O22	−1.4 (2)
N10A—C11A—C12A—N1A	4.5 (2)	C2—C3—C4—Cl4	−179.50 (14)
N10A—C11A—C12A—C13A	−175.62 (15)	C2—C3—C4—C5	0.9 (3)
C14A—C11A—C12A—N1A	−175.47 (15)	Cl4—C4—C5—Cl5	−1.6 (2)
C14A—C11A—C12A—C13A	4.4 (2)	Cl4—C4—C5—C6	178.04 (14)
N10A—C11A—C14A—C6A	177.13 (15)	C3—C4—C5—Cl5	178.02 (14)
N10A—C11A—C14A—C7A	−1.2 (2)	C3—C4—C5—C6	−2.4 (3)
C12A—C11A—C14A—C6A	−2.9 (2)	Cl5—C5—C6—C1	−179.20 (12)
C12A—C11A—C14A—C7A	178.83 (14)	C4—C5—C6—C1	1.2 (2)
N1A—C12A—C13A—C4A	−2.4 (2)		

### Hydrogen-bond geometry (Å, °)

**Table:**

*D*—H···*A*	*D*—H	H···*A*	*D*···*A*	*D*—H···*A*
N1A—H1A···O22	0.90 (2)	1.83 (2)	2.6926 (19)	158 (2)
N1A—H1A···N10A	0.90 (2)	2.38 (2)	2.749 (2)	104.3 (15)
O12—H12···O21	0.98 (3)	1.43 (3)	2.4054 (19)	179 (4)
C2A—H2A···O21	0.93	2.52	3.279 (2)	140
C3—H3···O22	0.93	2.26	2.647 (2)	104
C3A—H3A···O11^i^	0.93	2.44	3.355 (2)	168
C4A—H4A···O21^ii^	0.93	2.49	3.252 (2)	139
C6—H6···O11	0.93	2.29	2.668 (2)	103
C6A—H6A···O11^iii^	0.93	2.59	3.270 (2)	130

Symmetry codes: (i) −*x*+2, *y*+1/2, −*z*; (ii) −*x*+1, *y*+1/2, −*z*; (iii) *x*−2, *y*+1, *z*.
